# Depression and associated factors among primary caregivers of children and adolescents with mental illness in Addis Ababa, Ethiopia

**DOI:** 10.1186/s12888-019-2228-y

**Published:** 2019-08-13

**Authors:** Woredaw Minichil, Wondale Getinet, Habtamu Derajew, Sofia Seid

**Affiliations:** 1 Department of Psychiatry, College of Medicine and Health Sciences, University of Gondar, Gondar, Ethiopia; 2 Amanuel Mental Specialized Hospital, Addis Ababa, Ethiopia

**Keywords:** Depression, Primary caregiver, Mental illness, Children, Adolescent, Ethiopia

## Abstract

**Background:**

Mental illnesses among children and adolescents are under-recognized and under-treated problems. Depression is one of today’s all-too-silent health crises in caregivers. Although primary caregivers of children and adolescents with mental illness are more frequently depressed, little attention is being given to the problem in Ethiopia. Thus, this study aimed to assess prevalence of depression and associated factors among primary caregivers of children and adolescents with mental illness in Ethiopia.

**Methods:**

Institution-based cross-sectional study was conducted among primary caregivers of children and adolescents with mental illness in Ethiopia. Systematic random sampling was used to recruit a total of 416 study participants. Patient Health Questionnaire-9 was used to measure depression. After descriptive statistics was conducted, binary logistic regression was employed to carry out bivariate and multivariate analysis.

**Result:**

The overall prevalence of depression was 57.6% with 95% CI (53, 62.7). The prevalence of depression among female primary caregivers was 64.6% (*n* = 181). Female sex (AOR = 2.4, 95% CI: 1.18,4.89), duration of care > 5 years (AOR = 4.2, 95% CI: 2.02,8.70), absence of other caregiver (AOR = 2.7, 95% CI: 1.41,5.34), being mother (AOR = 3.9, 95% CI: 1.90,8.04), autistic spectrum disorder (ASD) (AOR = 4.7, 95% CI: 2.06,10.54) and attention deficit /hyperactivity disorder (ADHD) (AOR = 5.3, 95% CI: 2.14,13.23) diagnosis of children and adolescents and poor social support (AOR = 5.5, 95% CI: 2.04,15.02) were associated with depression.

**Conclusion:**

The prevalence of depression among primary caregivers of children and adolescents with mental illness attending treatment in St. Paul’s hospital millennium medical college (SPMMC) and Yekatit-12 hospital medical college (Y12HMC) was high. Therefore, it needs to screen and treat depression in primary caregivers of children and adolescents having follow-up at child and adolescent clinics especially for those primary caregivers who are female, mother, gave care for > five years, have no other caregiver, have children diagnosed with ASD and ADHD and have poor social support.

## Background

Mental illness is a psychiatric disease whose manifestations are characterized primarily by behavioral or psychological impairment of function, measured in terms of deviation from some normative concept; associated with distress or disease, not just an expected response to a particular event or limited to relations between a person and society [1]. Although neurodevelopmental disorders (e.g., Intellectual Disability (ID), Autism Spectrum Disorder (ASD), Attention Deficit/Hyperactivity Disorder (ADHD), Communication disorder etc), serious mental illnesses (e.g., schizophrenia, major depressive disorder, bipolar disorders etc) and other types of mental illnesses are categorized in different groups of mental as well as behavioral problems, they all are under the umbrella of mental illness [1, 2].

According to World Health Organization (WHO) report in 2001, mental health problems among children and adolescents have increased in recent years and they are predicted to increase by 50% in the year 2020 [3]. WHO estimates that 15–20% of children have disabilities worldwide; of which, 85% are in developing countries like Ethiopia [4]. In 2016, about 6.1 million U.S. children with age ranging from 2 to 17 years had ever received ADHD diagnosis [5]. As per 2011 Census of India, there were about 7.8 million children with disability in the below 19 year age group [6]. In a study conducted among children and adolescents in Northeast China, the overall prevalence of mental illness was 9.49%. Of these mental illnesses, anxiety disorders (6.06%), depression (1.32%), oppositional defiant disorder (1.21%) and attention-deficit hyperactivity disorder (0.84%) were the most common disorders [7]. From the total number of persons with disabilities in Ethiopia, 30.9% was under the group of 0–14 years of age and the percentage of intellectual disability was 6.5% [8]. Children with developmental delays have difficulties with major activities such as language, mobility, learning, self-help, and independent living [9].

Depression is a state of low mood and aversion to activity that can affect a person’s thoughts, behavior, feelings and sense of well-being. People with depressed mood can experience feeling of sadness, lack of interest, hopelessness, helplessness, worthlessness, guilty feeling, and irritability. They may also experience suicidal ideation, sleep disturbance and loss of appetite [1]. Compared to the general population, 40–60% of patients with major depressive disorder are exposed for greater chance of premature death, because depression is commonly associated with heart diseases, cancer and diabetes mellitus [10–12].

Depression is one of today’s all-too silent health crises and it was reported to be the most common disorder with the rate of 30–59% among caregivers [13]. Depression in caregivers of children and adolescents with different specific mental illness was estimated to vary from 19 to 79% [14–17]. Caregivers who are at risk for depression is almost three times higher than in general populations of similar age [18]. According to different study findings, women caregivers experience depression two to three times more than men in industrialized countries [19]. It was reported that level of depressive symptoms and mental health problems among caregivers of children and adolescents with mental disorders is higher than their non-care giving peers [20]. As compared to the general population, primary caregivers are more frequently depressed and anxious [21]. The symptoms of depression are commonly experienced by caregivers, which in turn, heighten their difficult situation [22]. Family caregivers are assumed to take the responsibility for the physical, emotional, medical and financial care of the sick relative. Those responsible caregivers of patients with mental illness, without proper preparation, knowledge, or support of health professionals, experience changes in their life [23]. Even though the positive and negative effects of caregiving are not always visible, the care tends to produce high levels of caregiver burden [24]. A child with a developmental delay may cause serious stress for the parents and can affect each member of the family who experiences a great amount of psychological distress [9]. Raising children with developmental disorders is a stressful event for a family because of the interrelated negative effects. Physical and psychological disabilities of children, because of their early onset and lifelong requirement for support and care, impose enormous social and economic burdens on the affected individuals, their families, and their communities at large [25]. With neglecting their physical as well as psychological health needs, caregivers usually give attention for the needs of those they care for [12]. An increased stress of caring a child with ID, ASD or ADHD plays a role in increased perceived stress, anxiety, and depression for caregivers [26]. Families of children with mental illness are more likely to experience divorce, and compromise their occupational competency and social mobility [27]. Presence of children with mental illness in the family carries a high risk for social and psychological burdens to their caregivers [14]. Depression among caregivers of children with mental illness has been associated with low income, being the only caregiver in the family and hyperactive/impulsive and combined types of ADHD [14], female sex, unemployment, primary education, college graduates, married status, and the age 31–45 years of caregivers [15, 16, 28, 29], being single, had child in the older age group (9–12 years) and had a female child [28, 30], giving care for more than five years [16] and absence of social support [28].

Despite a high impact of caring children and adolescents with mental illness on family caregivers, little attention is being paid to the problem as well as its physical, psychological and financial consequences in Ethiopia. Mental illnesses among children and adolescents are under-recognized and undertreated. Since the magnitude of depression and associated factors among primary caregivers of children and adolescents with mental illness has not been investigated and no statistical evidence including well-organized screening and treatment services for caregivers of children and adolescents in Ethiopia, this study hopefully fill the knowledge gap by generating the necessary information and serve as a source of direction for intervention. Finally, it will serve as a baseline data for further related studies. Thus, the main intention of this study was to investigate the prevalence and associated factors of depression among primary caregivers of children and adolescents with mental illness, Addis Ababa, Ethiopia.

Authors hypothesized that the prevalence of depression in primary caregivers of children and adolescents with mental illness is higher compared to caregivers of adults with mental illness and the general population.

## Methods

### Study design

An institution based cross-sectional study was conducted among primary caregivers of children and adolescents with mental illness attending outpatient department of child and adolescent psychiatry clinics.

### Study area and period

The study was conducted at SPHMMC and Y12HMC, located in Addis Ababa; the capital city of Ethiopia. Both hospitals are serving more than 5 million people in their catchment areas. Currently, these hospitals are teaching hospitals. They are providing organized child and adolescent psychiatric outpatient services. Child and adolescent psychiatric units have served for children and adolescents with mental illness five days per week. Around 15–20 children and adolescents were given follow-up service at outpatient department of the child and adolescent psychiatry clinic in each hospitals daily. The data were collected at psychiatry clinics from May 15–June 22, 2018.

### Sample size determination and sampling procedure

The sample size was determined by using the single population proportion formula with the assumptions of 56.2% prevalence of depressive disorder among caregivers of children with mental disorders from the study conducted in Kenya [16], 1.96 Z (standard normal distribution), 5% margin of error, 95% CI of certainty (alpha = 0.05), 10% non-response rate. Based on these assumptions, the total sample was 416.

We used the systematic random sampling technique to select 416 primary caregivers of children and adolescents with mental illness having follow-up for the treatment of their children and adolescents with mental illness. Primary caregivers who were 18 years and above, and those who provided care for at least six months were included in the study. Caregivers who had previous diagnosis of depression and treatment follow-up were excluded.

### Children and adolescents with mental illness

In the current study, children and adolescents with mental illness refers to children and adolescents who were below 18 years of age and who had a mental illness diagnosed in infancy, childhood and adolescence. The diagnosis was obtained from charts of children and adolescents.

### Outcome variable

The outcome variable was depression. It was measured by using an interviewer-administered patient health questionnaire (PHQ-9). PHQ-9 score ranges from 0 to 27. Each of the 9 items was scored from 0 (“not at all”) to 3 (“nearly every day”). A PHQ-9 score 10–14, 15–19 and 20–27 indicates moderate, moderately severe, and severe depression which requires immediate initiation of therapy [31]. Moreover, PHQ-9 has been validated in Ethiopian healthcare context with specificity and sensitivity of 67 and 86% respectively. A cut-off point of 10 and above has been used to screen depression [32]. In the current study, Cronbach’s alpha of the scale was 0.79.

### Independent variables

#### Socio-demographic factors

Socio-demographic variables included age, sex, religion, ethnicity, monthly income, employment status, marital status, number of children, educational status, duration of care-giving, caregiver’s relation to children and adolescents with mental illness.

#### Psychological and clinical factors

Perceived social support was among the psychological factors considered in this study, and it was assessed by using the Oslo 3-item social support scale which had a sum score ranges from 3 to 14 and had three broad categories. According to this category, respondents who scored 3–8, 9–11 and 12–14 were considered as having poor, moderate and strong social support respectively [33]. Chronic medical illness and family history of mental illness were assessed by using yes/no questions in primary caregivers of children and adolescents with mental illness.

#### Children and adolescents related factors

Age, sex and types of diagnoses were children and adolescents related factors.

#### Data collection procedures

Initially, all questionnaires were translated into local language (Amharic) before data collection and translated back by another bilingual expert in both English and Amharic to check its consistency. Data were collected from primary caregivers accompanied with children and adolescents with mental illness who had follow-up treatment service using interview technique at child and adolescent psychiatry clinic in SPHMMC and Y12HMC. Pre-test was done on a sample (5% of the total sample) of primary caregivers of children and adolescents with mental illnesses attending outpatient clinic at SPHMMC and Y12HMC prior to data collection was implemented. The findings of a pretest were not included in the main research report. Clinicians working in child and adolescent psychiatry clinics linked caregivers with data collectors, and the data collectors interviewed primary caregivers who were eligible. Training was given to four data collectors and two supervisors on basic data collection and interview techniques. Data quality and its completeness were monitored by supervisors at daily basis.

#### Data processing and analysis

Data were coded and entered into the Epi-data software version 3.1, and exported to Statistical Package for Social Science (SPSS, version 21) for analysis. After data cleaning, bivariate analysis was used to assess the associations between dependent and independent variables. Adjusted odds ratio with a 95% confidence interval was used to estimate the strength of the association. All variables associated with depression, with a *p*-value less than 0.2 in the bivariate logistic regression, were further analyzed using multivariate logistic regression analyses to control the confounding effects. Variables with a *P*-value less than 0.05 in the multivariate logistic regression were declared to be significantly associated with depression.

## Results

A total of 416 primary caregivers were invited to participate in the study. Of these, 408 responded to the interview which yielded a response rate of 98.1% that was higher than most of other related studies. The majority of the respondents, 280(68.6%), were female. The mean age of the respondents was 41.44 + 8.48(SD) years; 272(66.7%) were in the age category of 35–55 years; 198(48.5%) were Amhara by ethnicity; 239(58.6%) were married; 135 (33.1%) were employed in governmental institutions; 261(64.0%) were orthodox Christian; 341(83.6%) attended school in their life; of those, 134(39.3%) achieved college and above; 238(58.3%) were mothers; 244(59.8%) had given care for their children and adolescents with mental illness for greater than five years; 211(51.7%) had other caregivers; 233(57.1%) had poor social support; 78 (19.1) had chronic medical illness, and 57 (14.0) had family history of mental illness. Of the respondents, 80 (19.6%) used khat; 207(50.7) consumed alcohol, and a small number of the participants, 21 (5.1), smoked cigarettes (Table 1).

**Table 1:** Socio-demographic and clinical characteristics of primary caregivers of children and adolescents with mental illness attending child and adolescent psychiatric clinics in SPHMMC and Y12HMC, Addis Ababa, Ethiopia, 2018 (*n* = 408)

Variables	Category	Frequency (%)
Sex	Male	128 (31.4)
	Female	280 (68.6)
Age in year	18–35	115 (28.2)
	36–55	272 (66.7)
	> 55	21 (5.1)
Income (in ETB)	< 1539	114 (27.9)
	> 1539	294 (72.1)
Religion	Orthodox	261 (64.0)
	Muslim	70 (17.2)
	Protestant	63 (15.4)
	Others	14 (3.4)
Ethnicity	Amhara	198 (48.5)
	Oromo	100 (24.5)
	Tigrie	40 (9.8)
	Guragie	64 (15.7)
	Others	6 (1.5)
Marital status	Married	239 (58.6)
	Never married	28 (6.9)
	Divorced/separated	103 (25.2)
	Widow/er	38 (9.3)
Occupational status	Governmental employee	135 (33.1)
	NGO employee	139 (34.1)
	Merchant	48 (11.8)
	Farmer	13 (3.2)
	House wife	62 (15.2)
	Others	11 (2.7)
Residence	Urban	321 (78.7)
	Rural	87 (21.3)
Educational status	No formal education	67 (16.4)
	Primary	117 (28.7)
	Secondary	90 (22.1)
	College and above	134 (32.8)
Relationship with the patient	Father	155 (38.0)
	Mother	238 (58.3)
	Others	15 (3.7)
Duration of care	< 1 year	89 (21.8)
	1–5 years	75 (18.4)
	> 5 years	244 (59.8)
Presence of other caregiver	Absent	197 (48.3)
	Present	211 (51.7)
Perceived social support	Poor	233 (57.1)
	Moderate	127 (31.1)
	Strong	48 (11.8)
Khat chewing	Yes	80 (19.6)
	No	328 (80.4)
Alcohol drinking	Yes	207 (50.7)
	No	201 (49.3)
Cigarette smoking	Yes	21 (5.1)
	No	387 (94.9)
Chronic medical illness	Yes	78 (19.1)
	No	330 (80.9)
Family history of mental illness	Yes	57 (14.0)
	No	351 (86.0)

Others religion: Catholic, Jewish; Others ethnicity = Wolayta, Siltie, Sidama; others occupational status = Daily laborer, Retired; Others relationship = Aunt, Sister, Brother

Regarding to the children and adolescents with mental illness, about half (51.2%) were male; 202(49.5%) were under the age of 10 years, and 184(45.1%) were diagnosed with autistic spectrum disorder (Table 2).

**Table 2:** Socio-demographic and clinical characteristics of children and adolescents with mental illness attending child and adolescent psychiatric clinics in SPHMMC and Y12HMC, Addis Ababa, Ethiopia, 2018 (*n* = 408)

Variables	Category	Frequency	Percentage
Sex	Male	209	51.2
	Female	199	48.8
Age in year	< 10	202	49.5
	10–17	206	50.5
Type of diagnosis	ID	76	18.6
	ASD	184	45.1
	ADHD	92	22.5
	Others	56	13.7

Others: Epilepsy = 20, Schizophrenia = 13, Depression = 19, Post-traumatic stress disorder = 4

### Prevalence of depression

The prevalence of depression among primary caregivers of children and adolescents with mental illness was 57.6% (*n* = 235). Among respondents with depression, 181 (77%) were female.

### Factors associated with depression

To determine the association of independent variables with depression, bivariate and multivariate logistic regression analysis were carried out. In the bivariate analysis, factors including female sex, divorce, primary education and below, farming job, being mother, duration of care greater than five years, absence of another caregiver, chronic medical illness, children’ and adolescents’ ASD and ADHD diagnosis and poor social support were found to be significantly associated with depression at a *P*-value less than 0.2. These factors were entered into the multivariate logistic regression model to control confounding effects.

The results of multivariate analysis showed that female sex, being mother, duration of care > 5 years, absence of another caregiver, diagnosis of children and adolescents with ASD and ADHD and poor social support were significantly associated with depression at a *P*-value less than 0.05. The odds of developing depression were 2.4 times higher among female participants than males (AOR = 2.4, 95% CI: 1.18, 4.89). The odds of developing depression were 4 times higher among mother caregivers compared to fathers (AOR = 3.9, 95% CI: 1.90, 8.04). It was indicated that respondents who gave care for > 5 years were nearly 4 times more likely to exhibit depression compared to respondents who gave care for < 1 year (AOR = 4.2, 95% CI: 2.02,8.70). The odds of developing depression were around 3 times higher among respondents who had no other caregivers compared to those who had another caregivers (AOR = 2.7, 95% CI: 1.41,5.34). The likelihood of developing depression among those primary caregivers who had children and adolescents with autistic spectrum disorder (AOR = 4.7, 95% CI: 2.06, 10.54) and attention-deficit/hyperactivity disorder (AOR = 5.3, 95% CI: 2.14, 13.23) was 4.7 and 5.3 times higher compared to caregivers of children with intellectual disability respectively. The odds of developing depression was 5.5 times higher among those primary caregivers who had poor social support compared to those who had strong social support (AOR = 5.5, 95% CI: 2.04,15.02).

On the other hand, marital status, occupation, level of education and chronic medical illness were not statistically significant with depression in multivariate logistic regression (Table 3).

**Table 3:** Factors associated with depression among primary caregivers of children and adolescents with mental illness attending child and adolescent psychiatric clinics in SPHMMC and Y12HMC, Addis Ababa, Ethiopia, 2018 (*n* = 408)

Variables	Category	Depression		COR (95%CI)	AOR (95% CI)
		Yes	No		
Sex	Male	54	74	1.00	1.00
	Female	181	99	2.51 (1.63, 3.84)*	2.40 (1.18, 4.89)*
Marital status	Married	1251	114	1.00	1.00
	Single	3	15	0.79 (0.36,1.73)	0.75 (0.22, 2.53)
	Divorced	72	31	2.12 (1.3, 3.46)*	0.99 (0.48, 2.10)
	Widow/er	25	13	1.75 (0.86, 3.59)	0.67 (0.22, 2.04)
Occupational status	Go’tal employee	74	61	1.00	1.00
	NGO employee	77	62	1.02 (0.64, 1.65)	0.6 (0.28, 1.27)
	Merchant	29	19	1.26 (0.64, 2.46)	0.93 (0.31, 2.76)
	Farmer	10	3	2.75 (0.72, 10.43)*	1.06 (0.17, 6.71)
	House wife	37	25	1.22 (0.66, 2.25)	0.56 (0.20, 1.58)
	Others	8	3	2.2 (0.56, 8.65)	1.09 (0.17, 7.02)
Educational status	No education	51	16	4.05 (2.10, 7.82)*	2.46 (0.82, 7.41)
	Primary	84	33	3.24 (1.91, 5.49)*	1.83 (0.72, 4.62)
	Secondary	41	49	1.06 (0.62, 1.82)	0.47 (0.20, 1.09)
	>College	59	75	1.00	1.00
Relationship with patient	Father	67	88	1.00	1.00
	Mother	162	76	2.80 (1.84, 4.26)*	3.91 (1.90, 8.04)*
	Others	6	9	0.88 (0.3, 2.58)	1.51 (0.28, 8.14)
Duration of care	< 1 year	39	50	1.00	1.00
	1–5 years	39	36	1.39 (0.75, 2.57)	2.45 (0.98, 6.11)
	> 5 years	157	87	2.31 (1.41, 3.79)*	4.19 (2.02, 8.70)*
Other caregiver	Absent	145	52	3.75 (2.47, 5.70)*	2.74 (1.41, 5.34)*
	Present	90	121	1.00	1.00
Medical illness	No	196	134	1.00	1.00
	Yes	39	39	0.68 (0.42, 1.12)*	0.63 (0.31, 1.28)
Type of diagnosis	ID	26	50	1.00	1.00
	ASD	137	72	3.66 (2.12, 6.36)*	4.66 (2.06, 10.54)*
	ADHD	64	43	2.86 (1.55, 5.28)*	5.32 (2.14, 13.23)*
	Others	8	8	1.92(0.65, 5.71)	3.00(0.59,15.44)
Perceived social support	Poor	181	52	6.96 (3.55, 13.67)*	5.54 (2.04, 15.02)*
	Moderate	38	89	0.85 (0.420, 1.737)	0.58 (0.22, 1.50)
	Strong	16	32	1.00	1.00

NOTE: Model fitness: Chi-square = 8.82; df = 8; sig (*p*-value) = 0.36; *statistically significant at *P* < 0.05

## Discussion

Having children and adolescents with mental illness in the family has a high risk for psychological and social burdens to the caregivers [14]. Depression is among the largest single causes of disability worldwide [34]. The main reason for assessing depression among caregivers in clinical practice is to ensure that early recognizing, screening and treating depressive symptoms that focus on the caregivers’ risk factors. Hence, this institution-based cross-sectional study was conducted to assess depression and associated factors by using PHQ-9 and social support was also assessed using Oslo-3 social support scale.

The prevalence of depression in Ethiopia varied from 0.6% among women in a community-based study using Composite International Diagnostic Interview (CIDI) [35] to 23.6% among students identified with PHQ [36]. Its pooled prevalence of eight studies was also 11% [37] which is much less than this study finding. The current study found that the prevalence of depression was 57.6% with 95% CI (53, 62.7) among primary caregivers of children and adolescents with mental illness, which was lower than a cross-sectional study conducted in Muscat 65% [14], and Kenya 79% [15]. This difference might be attributed to: socio-cultural variation i.e., perception of caregivers’ losses and how they interpret their distress differs across cultures and social integrity, and difference in sample size in Muscat, and it was done on caregivers of children with only a specific disorder i.e., ADHD, time-point the studies conducted, sample size and tools difference i.e., long-term study, small sample size and BDI scale in Kenya. Conversely, the finding of this study was higher than studies conducted in US 37% [38], Mexico 22.7% [39] and Texas 19% [17]. This discrepancy might be due to variation in study subjects who were caregivers of children younger than 5 years old in US, BDI measurement tool in Mexico and sample size difference in which 110 caregivers were studied in Texas. On the other hand, the prevalence of depression in the current study was consistent with reports of studies on caregivers of children with neurological diseases in Spain 53% [40], mental health needs in Midwestern state 57.4% [41] and mental disorders in Kenya 56.2% [16].

Regarding the factors associated with depression in the current study, the odds of developing depression was 2.4 times higher among female participants than males. This association was consistent with another similar study conducted in Kenya and Pakistan [15, 29]. The possible reason might be females experiencing more caregiving stressors like higher social expectations and lower social support [16]. The fact also indicates that females are responsible for the emotional care of the children and to take up the role of the caregiver. Another suggested reason for this association might also be sex hormones having some influences on depression. So females who are responsible for caregiving for children and adolescents with mental illness could be disturbed with their health condition and end up with different psychiatric problems such as depression. Another factor significantly associated with depression was being mother of children and adolescents with mental illness. The odds of developing depression were 4 times higher among mothers compared to fathers. This was supported by studies conducted in Kenya [15]. The possible reason might be mothers’ reaction to caregiving with a greater tendency to become distressed and to feel burdened by caregiving than fathers. Being mother increased subjective internalized strain of the caregiver [16, 42]. This leads mothers to have more negative feelings like worry, guilt, sadness and fatigue regarding their child with a mental health problem. Giving care for > five years was significantly associated factor with depression. Respondents who gave care for > 5 years were 4 times more likely to exhibit depression than those who gave care for < 1 year which was in agreement with other studies conducted in Kenya and Texas [15, 43]. The likely reason could be caregiving stressors that can increase and persist the caregiver’s depressive symptomatology [16, 43]. In general, as the duration of care increases, the level of depressive symptoms becomes worse. The absence of another caregiver for children and adolescents with mental illness at home was significantly associated with depression. The odds of developing depression were around 3 times higher among respondents who had no other caregivers than those who had another caregiver. This was in line with the study done in Muscat [14]. The possible reason might be the burden of caregiving and restriction from social involvement that leads to feeling of sadness and loneliness. It is also not surprising that the unavailability of another caregiver in the family to share the responsibility of caregiving leads to depression. Diagnosis with ASD and ADHD was also found to be significantly associated with depression. The likelihood of having depression was 4.7 and 5.3 times higher among those primary caregivers who had children and adolescents with ASD and ADHD compared to caregivers of children with intellectual disability respectively. This is consistent with the findings in Muscat and Taiwan [14, 44]. The possible reason may be the functional dependence and severity of maladaptive behaviors in ASD and ADHD compared to ID that increases the caregiving burden and pessimism [45]. Another reason also might be higher levels of externalization behaviour that are related to the level of caregiver distress [46]. Having poor perceived social support was also another predictor of depression in primary caregivers of children and adolescents with mental illness The odds of developing depression among those primary caregivers who have poor social support was 5.5 times higher as compared to those who have strong social support. This view was supported by the study conducted in Saudi Arabia [28]. The possible reason is that lack of social support for caregivers of children and adolescents with mental illness revealed that caregivers with poor support networks reported higher levels of depression. Low level of social support is also the most powerful predictors of depression in caregivers [47].

Since we have employed face to face interview, the study might be affected by social desirability bias. The study design was also a cross-sectional; so that it was difficult to establish a temporal relationship between depression and significantly associated factors. Comorbid diagnosis among children and adolescents was not considered in the study. In addition, the tool we have used to assess social support was not validated in the Ethiopian context.

## Conclusion

The prevalence of depression was found to be high. This may have a marked effect on the physical as well as mental health of children and adolescents who are under their care. Female sex, being mother, duration of care > five years, absence of another caregiver in the family, caring children and adolescents diagnosed with ASD and ADHD and poor social support were significantly associated with depression. The current finding showed important evidences and actions to be taken for the existing conditions in developing countries including Ethiopia. Therefore, early screening and treating primary caregivers is very important to reduce the incidence of depression. Special attention should be given for primary caregivers providing care for a longer period; caregivers of children and adolescents with ASD and ADHD; those who are female and mother; those who have no other caregivers helping them in care and those having poor social support.

## Data Availability

All data included in the manuscript has been included in the form of tables. The non-identified raw data is not publicly available but it can be requested from the corresponding author after providing the necessary justification for request; Woredaw Minichil: woredawm21@gmail.com

## Abbreviations

ADHD: Attention Deficit/Hyperactivity Disorder
AOR: Adjusted odd ratio
ASD: Autism spectrum disorder
CIDI: Composite international diagnostic interview
ID: Intellectual disability
PHQ-9: Patient health questionnaire-9
SPHMMC: St. Paul’s Hospital Millennium Medical College
SPSS: Statistical package for social science
Y12HMC: Yekatit-12 Hospital Medical College
